# Historical Sketch of Some of the London Hospitals.—II

**Published:** 1904-02-20

**Authors:** J. E. R. Stephens


### HOSPITAL ADMINISTRATION.

### CONSTRUCTION AND ECONOMICS.

### HISTORICAL SKETCH OF SOME OF THE LONDON HOSPITALS.—II.

By J. E. R. STEPHENS.

(*Continued from page* 345).

#### ST. THOMAS'S HOSPITAL.

IN 1213 Thomas, Prior of Bermondsey, erected a building for the reception of converts and poor children on a piece of ground belonging to the cellarer of his priory, adjoining the wall of his monastery, giving him a compensation of 10s. 4d. a year. He called it *The Almery*. In 1535 it was surrendered to King Henry VIII. After the dissolution the hospital was neglected, and the buildings became ruinous; but in 1552 Ridley, Bishop of London, by a well-timed sermon before the young King, awakened the benevolence of his disposition; the King consulted with him how he should commence some great charitable institution, and by his advice addressed a letter to the Mayor and Corporation of London announcing his intention and requiring their advice. After some consultations, at which the Bishop assisted, three different institutions were suggested, which at length produced Christ's Hospital for the education of youths, Bridewell for the poor and correcting the profligate, and this of St. Thomas for the lame and diseased. The King highly approved of these plans, and steps were immediately taken for establishing such foundations.

The City purchased St. Thomas's of the King, and in July, 1552 began to repair and enlarge it; and so diligent were they in the work that expending about £11,000 thereon, in the November following they received into it no less than 260 poor infirm persons. The King was so well pleased with what they had done, that on June 26, 1553, he granted them a Charter of Incorporation for this foundation. In this year the City published an account, by which it appeared that they had expended on this and Christ's Hospital, in repairs and furniture, £2,479 10s. 10d.

In the British Museum, Sloanian Manuscript 6,277, is an extract from the Proceedings at a Court held by the Governors April 6, 1579, stating that "for divers good causes moving this Court, and that the rents as well of those manors, lands, etc., wherewith it pleased our late Sovereign King Edward VI. moved with compassion of the state of the poor, to endow the Mayor etc. with all, which now belong to the Houses of St. Thomas's Hospital, as of all other lands, etc., which may be given or purchased to the use of this House, may be truly employed for relief of the poor, according to the godly meaning of the late King and other benefactors; and that such manors, lands, etc., as to be fitted to farm for provision of grain to this house, may be reserved for that purpose, that good grain may be found by our tenants, and that the rents of the residue may be increased in godly sort, according to the times for that the yearly expenses do exceed the yearly revenues, *communibus annis*, £300 a year at least. Therefore by general consent of the Governors, it is ordered that there shall be no lease or grant in reversion, or in possession, till one year, or two at most, of the expiration of the old lease, and that but for 21 years, or under, and that not to any Governor or to his use, nor of more land than in the occupation of the lessee at such time. But building leases may be granted of old decayed houses or pieces of land in London or the suburbs."

It was ordered that a minute-book of the proceedings of the Governors was to be kept. If a lessee shall underlet, the actual occupier to have the option of renewal, after him such person as will give most, and undertake to reside. All estates to be surveyed by the surveyor, sometimes by the treasurer, before letting. Tenants are bound to all repairs. The beef of the poor is to be bought by weight without bones, and the candles taken in exchange for tallow. For every quarter of wheat delivered at the waterside our baker delivers to the hospital 36 dozen and 18 loaves of bread without any vantage, of the goodness of London wheaten bread, each loaf to weigh 16 ounces.

The hospital suffered greatly in its possessions, though not in its own buildings, by the fires in 1676, 1681, and 1689. The building being from its age greatly decayed, a subscription was set on foot to rebuild and enlarge it, to which the Governors themselves contributed liberally, and amongst them Sir Robert Clayton stands conspicuous. He was of Marden, in the county of Surrey, and besides what he gave to this hospital in his lifetime, he by his will gave to it £2,300. He died on July 16th 1707. The first stone of the new building was laid by Sir John Fleet, who was Lord Mayor in 1692.

In 1613 there were in this hospital 780 persons, and those under care 205. In 1629 there were cured 843, buried out of the hospital 209. In the Report of 1802 it is stated that "in the last year there had been cured and discharged, of wounded, sick and diseased persons, 2,910 of in-patients, many of whom have been relieved with money and necessaries to accommodate them in their journeys to their own habitations. Out-patients 4,414. Buried after much charge in sickness 214; remaining under cure in-patients 402, out-patients 176. So that there are and have been in the last year under cure in the hospital, destitute of other proper cure 8,116."

When King Charles II. seized the charters of the City of London he issued a Commission to remove from St. Thomas's Hospital all those who did not approve of the arbitrary measures of the time. Several of the governors, and Mr. Hughes, the chaplain of the Hospitallers, were ejected. On the Revolution the charter of the City was restored, the intruding governors and the hospitaller were removed, and the former ones replaced. By the charter of King Edward the City was to support two fit and convenient ministers to celebrate divine service and administer the sacrament to the poor and to the parishioners of St. Thomas. By the rules for the government of the Royal Hospitals one of these was called the Hospitaller, and his office was of a mixed nature; he was to visit the poor in extremes and sickness, but he was also to receive of the steward the provisions, enter them in a book, and deliver as much as was needful to the cook; he was to receive the persons applying for admission, and to present to the governors such as were cured. Afterwards he was eased of all burdens not spiritual.

In the Library of the London Institution is "an appeal from a few of the meanest and most inconsiderable of the governors of St. Thomas's Hospital, to the generality of the noblest, wisest, wealthiest, and best of them, occasioned by some great injustice and high affronts which two or three bigots of that hospital have endeavoured to put upon Dr. Samuel Rolls the minister thereof." There is no date, but it seems to be of the time of Charles II. He describes himself as an ancient divine, a man who had been a Doctor in Divinity for twenty-four years, one of his Majesty's Chaplains-in-Ordinary, and the faithful minister of this hospital for fifteen years past, who has prayed and preached in the house three times a week, sometimes more, besides visiting the sick that desired it of him, and many others that never sent to him, two hours at a time. He then reproaches the Governors for not presenting him to the Parish Church. "Is this my recompense for having done you four times as much service, for £40 a year, as the young Minister of the Parish is required to do for four score; to prefer one who for age might have been my son—a bachelor, before one who had a wife, children and grandchildren—who lost a child in attending on the plague—lay for dead two or three months by an unhappy stroke received there—affront me before a whole Court, and talk of turning me out with infamy?"

The building having fallen into disrepair was entirely rebuilt in 1701–6 by public subscription. In 1862 the South Eastern Railway, requiring a portion of the site for their branch line to Charing Cross, gave, by award, £296,000 for the building and ground. The present hospital, which was rebuilt on 8½ acres of ground recovered from the river by the construction of the Albert Embankment, lies between Westminster Bridge and Lambeth Palace, and was commenced in 1868 and opened by the Queen in June, 1871. The building, of red brick, was constructed by Mr. H. Currey, at a cost of £500,000, of which £90,000 was paid to the Metropolitan Board for the site.

### GUY'S HOSPITAL.

This great and extensive foundation was the work of one man, Mr. Thomas Guy, a citizen and stationer of London, who began the building in his lifetime and lived to see it covered in, though not completely finished. He, however, made so good a choice of trustees that his design was fully carried into execution, and that without any loss of time. He was born in Fair Street, Horsleydown, in 1644 or 1645, being the son of Thomas Guy, a lighterman and dealer in coals, on whose death, about 1652, his mother returned with her three children to Tamworth, the place of her nativity, where she married a second time. On September 3rd, 1660, Thomas, the eldest son, was bound apprentice to John Clarke, jun., bookbinder and stationer in Mercers' Hall Porch, Cheapside, and on the expiration of his service he opened a shop at the corner house between Cornhill and Lombard Street, known as the Oxford Arms, with a stock of about £200. On October 7th, 1668, he was admitted a freeman of the Stationers' Company, of the City on the 14th, and on October 6th, 1673, was received into the Livery. His first successes in trade were owing to the great demand for Bibles printed in Holland, in which he was a considerable dealer, till the importation of them was prohibited; after which, having contracted with the University of Oxford for their privilege of printing the same, and furnished himself with types from Holland for that purpose, he carried on the trade in this branch for several years with very great advantage.

But whatever foundation he might have laid for his future acquisitions, in the ordinary way of his occupation, the immense wealth of which he died possessed arose chiefly from the great profits then made of "Seamen's Tickets," by purchasing them at a large discount, and subscribing them afterwards in the South Sea Company, established in 1710 for the purpose of discharging them, and giving an interest of 6 per cent. In this he was so early and considerable a trader, that in 1720 he was possessed of £45,500 stock, by prudently disposing of which when it bore a considerably advanced price, he acquired that amazing sum of which he died possessed.

Much undeserved obloquy has been thrown on his memory for his dealing in Sailors' Tickets and South Sea stock; but it should be remembered that as to the former, the blame of their tickets being brought to market belongs to the Government of that time, who, instead of paying them in money as they ought to have done, gave them tickets payable at a future time, which to them who wanted money for present purposes were useless unless they could procure cash for them, and this could not be obtained but at a discount; a method of raising a present supply which they would be sure to adopt, regardless of the loss sustained by it. As to South Sea stock, it does not appear that Mr. Guy had any hand in framing or conducting that abominable fraud; he bought the stock, and had the good judgment to sell it in time to avail himself of the rise. Much of his money, however, was acquired in the course of successful industry through a long life, and much arose through frugality and self-denial, which was probably necessary at his outset in life, and which became a habit not to be broken off.

Moreover, he was not inattentive to the wants of his relations in his lifetime, nor was all his wealth hoarded up to be distributed after he could no longer use it himself. By his will, it appears that he had lent money to some of his relations and had granted annuities to others. In his lifetime he had been a liberal benefactor to St. Thomas's Hospital, to which he had given £100 a year for eleven years preceding his own foundation, and had built three wards at his own expense, and had laid out £3,000 on improvements of the building. He had also founded an almshouse for 14 poor people in his mother's native town of Tamworth, which he represented in several Parliaments where he showed himself a zealous supporter of liberty and the rights of his fellow-subjects. By his will, he did not forget his relations, even those of a remote degree; to those who may be presumed to be advanced in years he left annuities from £10 to (in one instance) £200 a year, to the amount in all of £810 a year; to those who were younger, extending to grandchildren of his uncles and aunts, he left stock in the funds, mostly in sums of £1,000 each, to the extent of more than £64,000. By his will he gave to Christ's Hospital a perpetual annuity of £400 to receive four children yearly on the nomination of his trustees, a preference being given to any of his relations who might offer themselves. He also endowed his almshouse at Tamworth, and left £1,000 to discharge poor prisoners in London, Middlesex, and Surrey, at £5 each, and another £1,000 to be distributed amongst poor housekeepers at the discretion of his executors.

Having formed his plan, at the age of 76, he procured from the Governors of St. Thomas's Hospital a lease of a spacious piece of their ground, for a term of 999 years at a rent of £30 per annum. The following, from the Minutes of St. Thomas's Hospital, 1721, explains the foundation of Guy's Hospital: "Our worthy Governor and Benefactor, Thomas Guy, Esq., intending to found and erect a hospital for incurables within the close of this hospital, we have agreed to grant a lease to him . . . of several parcels of ground, which are purchased by the said Thomas Guy, or in trust for him, for 1,000 years, at £30 per annum, tax free." The word "incurables," here meaning also cases capable of cure, but requiring time, which cases Guy's was intended to receive at once, or from Thomas's and other hospitals. In the discretion of the governors its use soon became as other hospitals. This partial disregard of the original intention so disgusted Dr. Mead that he declined the honour of being president. The building of the hospital cost £18,793 16s. 1d., and the endowment amounted to £219,499 0s. 4d. A little more than a week after Guy's death the hospital was opened, and on Thursday, January 6th, 1725, 60 patients were admitted.

Guy's Hospital, as originally built, was a large and airy brick structure, consisting, besides the street front and two wings extending from it, of two spacious quadrangles in the rear, and providing accommodation, according to the terms of the endowment, for 400 patients. But a bequest by William Hunt enabled it to be enlarged by the construction of an additional wing for 300 more patients. In 1851-52 a new wing was erected and large additions made under R. M. Hawkins, architect, at a cost of £28,435.

